# Slippery when wet: The effect of a novel zwitterionic coating on silicone ureteral stents

**DOI:** 10.1007/s00240-026-02070-6

**Published:** 2026-10-07

**Authors:** Ye Jean Lim, Ye Na Diana Lim, Connor A. Church, Elijah Haynal, Elizabeth A. Baldwin, D. Duane Baldwin

**Affiliations:** Department of Urology, Loma Linda University Health, 11234 Anderson Street, Loma Linda, CA 92354 USA

**Keywords:** Friction, Ureter, Stents, Lubricants, Silicone

## Abstract

Silicone ureteral stents provide anti-encrustation properties and patient comfort but are more difficult to insert and remove due to increased surface friction. Applying zwitterionic coatings to silicone stents may reduce friction and facilitate stent placement and removal. This study compared the frictional forces of zwitterionic-coated silicone stents with commercial stents using benchtop models. The static and kinetic frictional forces of five ureteral stents were measured: bare silicone stent without coating (BS), single outer-coated zwitterionic silicone stent (Z1), double-coated (inner and outer) zwitterionic silicone stent (Z2), Black Silicone Filiform stent (“Black Beauty”) (BB), and Universa Soft stent (US). Friction was assessed with a submerged guidewire model to isolate stent–guidewire friction and a porcine ureteral model to simulate intraoperative stent insertion and removal. Five trials per stent were performed using a force gauge integrated with an Arduino-controlled motor system. Statistical significance was assessed using linear mixed-effects models with stent type and trial as fixed effects and ureter specimen as a random intercept. Pairwise comparisons were adjusted using the Holm method. In the submerged guidewire model, Z2 had 99%, 47%, and 40% lower kinetic friction than BS, BB, and US, respectively. During stent insertion, Z2 had 71%, 43%, and 44% lower kinetic friction than BS, BB, and US, respectively. During stent removal, Z2 had 92%, 58%, and 60% lower kinetic friction than BS, BB, and US, respectively. Linear mixed-effects models demonstrated a significant effect of stent type for all six friction conditions (all *p* < 0.001). The double-coated (inner and outer) zwitterionic silicone stent (Z2) demonstrated the lowest frictional forces across all testing conditions. Zwitterionic surface modification may therefore reduce friction during silicone stent placement and removal.

## Introduction

Ureteral stents are widely used to maintain urinary drainage in a range of benign and malignant urologic conditions [1]. However, indwelling stents and their placement and removal frequently cause urinary symptoms, hematuria, stent-related pain, and reduced quality of life in over 80% of patients [2]. Beyond discomfort, stent-related complications such as infection, biofilm formation, and encrustation remain clinically significant [1, 3]. These adverse events are influenced by stent material, surface chemistry and wettability, and frictional behavior, all of which contribute to protein adsorption, bacterial adhesion, and crystal deposition [3, 4]. Encrustation risk increases with dwell time and can lead to serious complications, including obstruction and difficult stent removal [3].

Silicone ureteral stents have gained interest due to their favorable biocompatibility, softness, and lower rates of encrustation compared with many polymer-based alternatives, with some studies reporting improved patient comfort and tolerance [5–7]. However, despite these advantages, silicone stents have not been widely adopted because their high intrinsic coefficient of friction can increase insertion difficulty during guidewire-assisted placement and removal, particularly in narrow or tortuous ureters [5–7].

Among emerging surface technologies, zwitterionic polymer coatings, which contain an equal number of positive and negative charges, have gained attention for their ability to form a strongly bound hydration layer that resists protein adsorption, bacterial adhesion, and biofilm formation [8]. Application of zwitterionic coatings has been shown to reduce biofilm formation by approximately 80% compared with unmodified urinary catheters [9]. More recently, zwitterionic multifunctional coatings have demonstrated substantial reductions in bacterial colonization in dynamic urine models, with up to 90% reduction in biofilm formation compared with unmodified control devices [10]. However, past attempts at coating silicone stents with zwitterions were not commercially viable. Recently, a strategy for applying a zwitterionic coating to silicone stents has been developed [11]. Nonetheless, the static (*F*_*s*_) and kinetic (*F*_*k*_) frictional behavior of zwitterionic-coated silicone ureteral stents under clinically relevant, tissue-mimicking conditions have not been studied. We hypothesize that this zwitterionic coating on silicone stents could reduce frictional forces and potentially simplify stent insertion and removal.

Therefore, this study aims to directly quantify and compare *F*_*s*_ and *F*_*k*_ for zwitterionic-coated silicone ureteral stents versus widely used commercial stents using both a submerged guidewire model and an ex vivo porcine ureteral model, to determine whether zwitterionic coatings can overcome silicone’s intrinsic frictional limitations.

## Methods

In this benchtop study, five ureteral stents with different coating configurations were evaluated, including a bare silicone stent without coating (BS), a single outer-coated zwitterionic silicone stent (Z1), and a double-coated (inner and outer) zwitterionic silicone stent (Z2) (Silq Technologies Corporation, Sunny Isles Beach, USA), as well as a Black Silicone Filiform Stent (“Black Beauty”) (BB) and a Universa Soft stent (US) (Cook Medical Inc., Bloomington, USA). Each stent type was tested on three experimental models: (1) submerged guidewire friction trial, (2) porcine ureteral removal trial, and (3) porcine ureteral insertion trial. For each stent type, five trials were performed to measure *F*_*s*_ and *F*_*k*_. Three independent porcine ureteral specimens were used for friction testing.

**Fig. 1:**
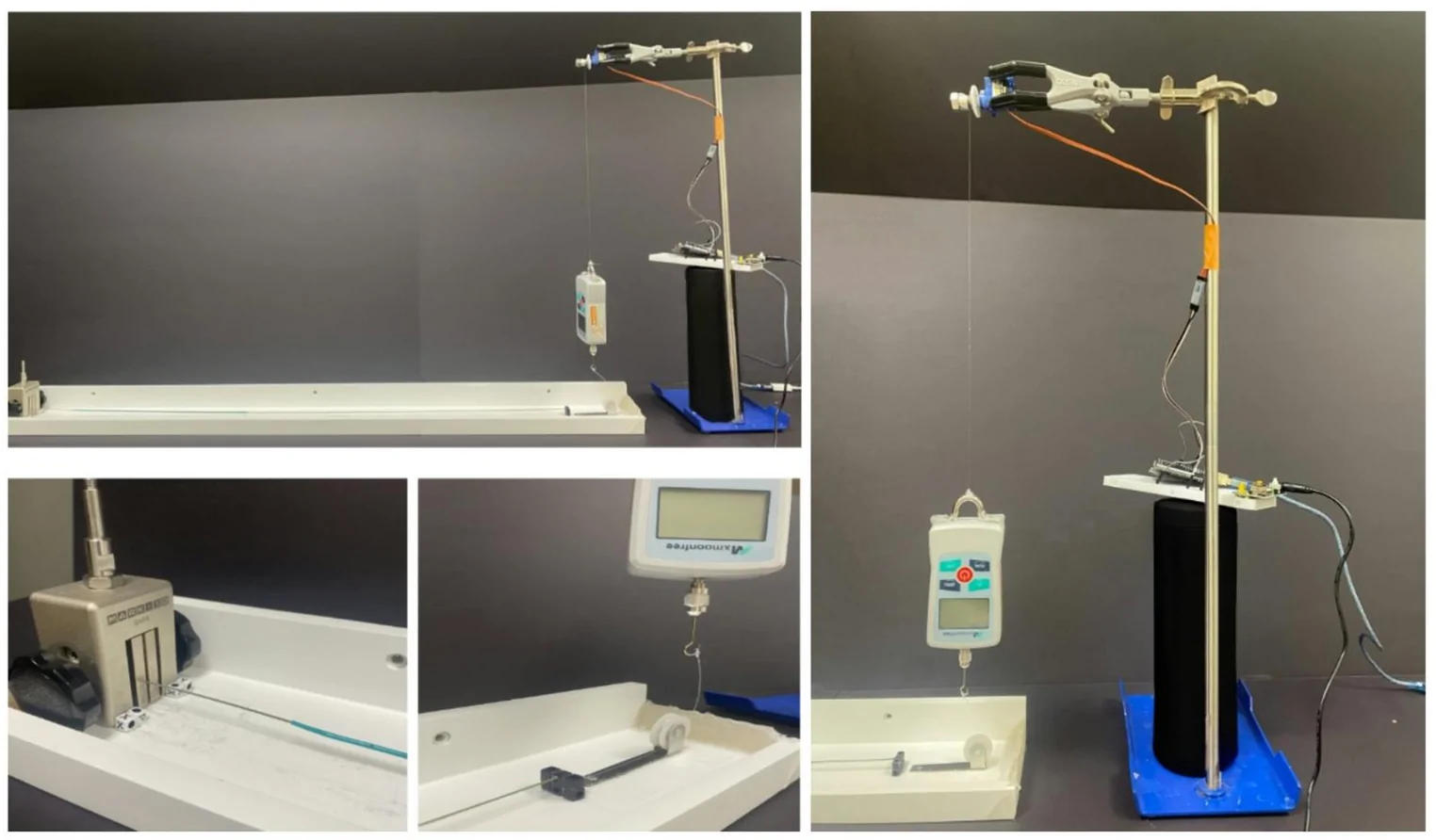
Submerged guidewire experimental model used to measure static and kinetic friction of ureteral stents. The setup includes a secured guidewire submerged in water, a nylon monofilament–pulley system attached to the stent, and an Arduino-controlled motor that vertically drives a digital force gauge to produce a horizontal pulling force

In the first experimental model, a submerged guidewire was used to isolate and measure the frictional forces of a stent being advanced over a standard clinical 0.038-in × 150-cm guidewire (Boston Scientific Corporation, Marlborough, USA), which was secured horizontally in the air to eliminate friction between the guidewire and the underlying tray. A stent was inserted over the guidewire at one end of the setup. A clear nylon monofilament line (Acejoz, Shenzhen, China) was then attached to the stent. This line was routed through a separate fixed bead to ensure a horizontal pulling force on the stent, then extended over a low-friction pulley positioned at the edge of the setup and was connected to the hook of the digital force gauge (0.001–5 N; Mxmoonfree, China) (Fig. 1). The force gauge was positioned vertically and linked via a second nylon monofilament line to a wheel driven by an Arduino-controlled motor. The motor was programmed to pull the force gauge vertically upward, thereby transmitting a horizontal pulling force to the stent against the fixed guidewire. Lastly, the tray was filled with water to ensure that both the guidewire and the stent were completely submerged throughout the trials (Fig. 1).

In the second and third experimental models, three independent porcine ureteral specimens (Specimens 1–3) were used to simulate intraoperative stent removal and insertion. Each renal unit was evaluated with all five stent types, and the order of stent testing within each renal unit was determined using computer-generated randomization. Because three specimens were available for five trials per stent, repeated trials were performed within the same specimens using the same ureteral segment. For the final ten trials, the ureter specimen was selected using computer-generated randomization. Institutional animal care and use committee (IACUC) approval was waived because porcine tissues were obtained post-mortem from animals used for food production at a licensed abattoir, and no animals were euthanized specifically for this research. The porcine urinary tracts (kidney, ureter, and bladder) were dissected to preserve the continuity of the urinary tract while minimizing tissue trauma. The kidney was coronally sectioned to expose the renal pelvis and ureteropelvic junction (UPJ), and the bladder was incised to reveal the ureterovesical junction (UVJ).

**Fig. 2:**
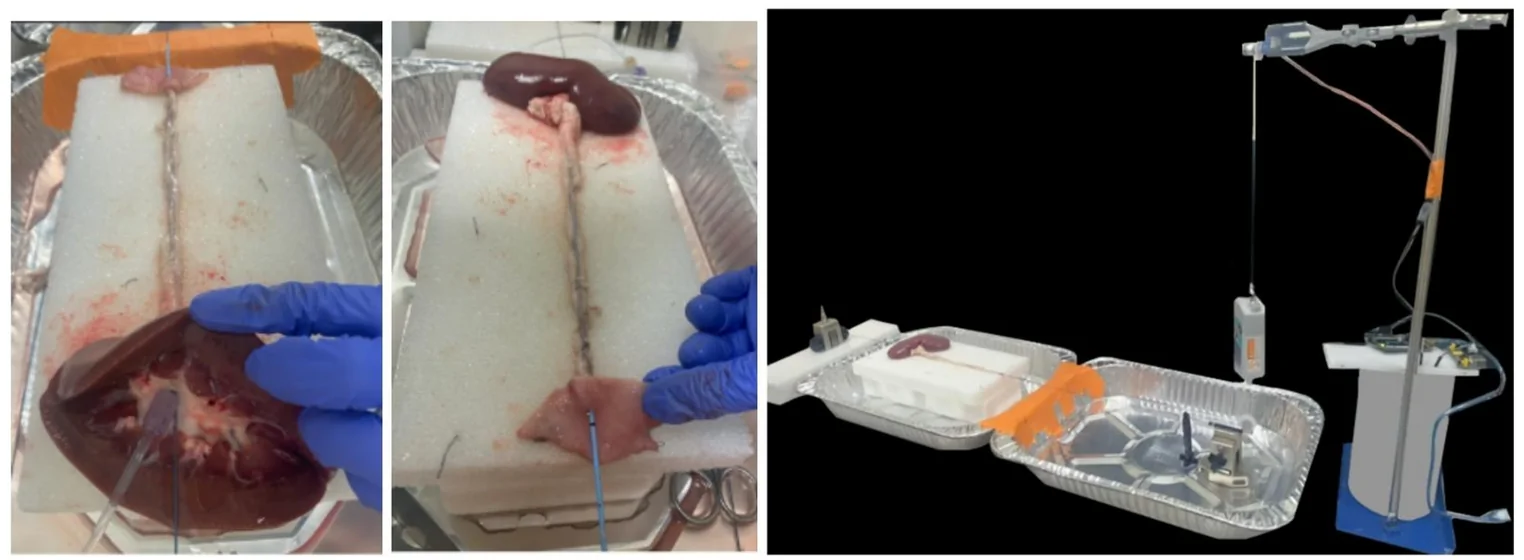
Porcine ureteral experimental model used to measure stent friction during removal. Close-up views show the exposed renal pelvis and UPJ with continuous irrigation (left), and the incised bladder exposing the UVJ (middle). The full setup includes the mounted force-gauge and pulley system used to pull the stent over an internal guidewire (right)

The second experimental model used two aluminum trays. The primary tray housed the porcine urinary tract, while an adjacent tray contained the guidewire-securing bead and pulley system. A guidewire was introduced through the renal pelvis, passed through the UPJ, advanced along the ureter, and exited through the UVJ into the bladder. The stent was then advanced into the ureter over the guidewire using a pusher. The kidney and bladder were secured to a foam leveler with pushpins to stabilize the system (Fig. 2).

To maintain tissue moisture throughout the experiment, a continuous irrigation system bathed the urinary tract with water. The nylon monofilament line attached to the stent was routed through the pulley system and connected to the digital force gauge, as previously described, ensuring horizontal force application (Fig. 2). The Arduino-controlled motor system provided precise vertical displacement of the force gauge, thereby measuring the frictional force exerted on the stent by the ureteral tissue and the internal guidewire.

**Fig. 3:**
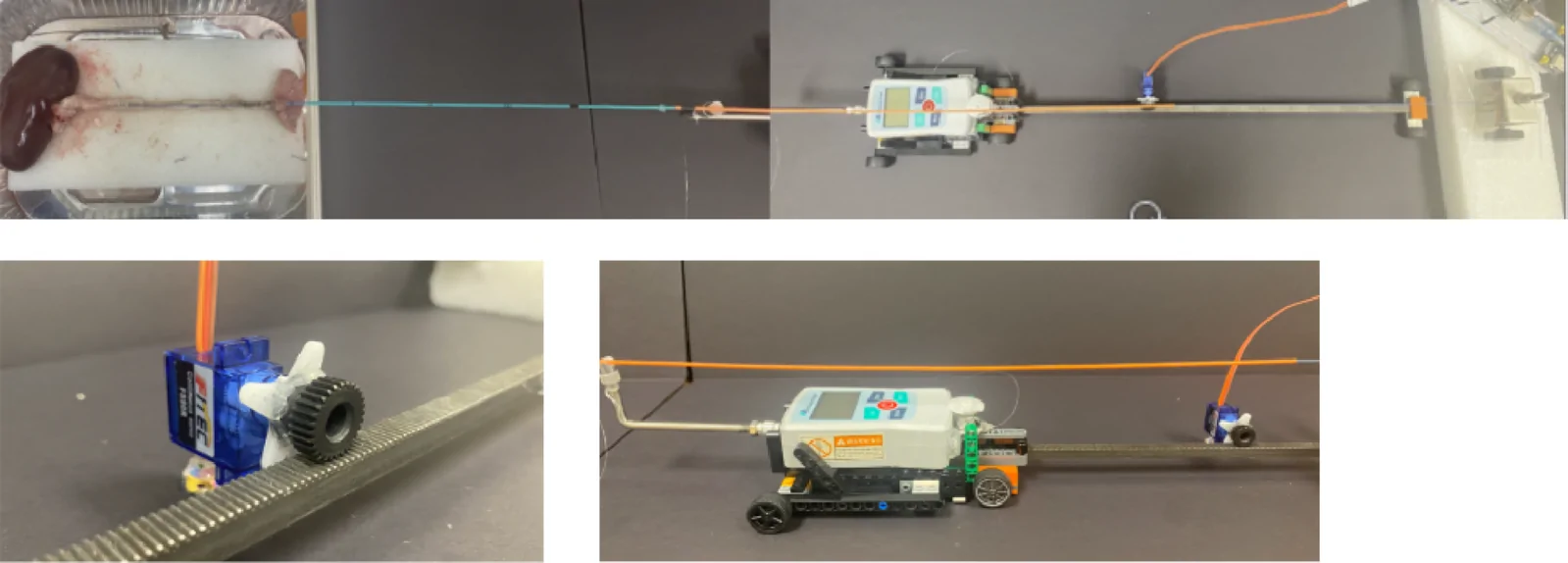
Porcine ureteral experimental model used to measure stent friction during insertion. The top panel shows the overall setup with the guidewire suspended above the porcine ureter and the stent positioned on the pusher. The bottom left panel provides a close-up of the motor-driven gear-and-rack assembly that advances the force gauge and pusher. The bottom right panel shows the force gauge mounted on the linear track used to push the stent through the ureter

In the third experimental model, a porcine ureter was used to simulate intraoperative stent insertion. The experimental setup consisted of a shallow tray containing the intact porcine urinary tract, with the guidewire secured to the tray at one end and attached to an elevated clamp at the other end to permit rapid release. The ureteral stent and pusher were sequentially threaded onto the guidewire outside the porcine urinary tract (Fig. 3).

The digital force gauge was mounted on a custom wheel assembly incorporating a carbon steel gear rack and mating gear wheel (Uxcell, Hong Kong, China). The assembly moved linearly beneath the elevated guidewire when driven by a stationary Arduino motor coupled to the gear wheel, providing precise and controlled linear displacement of the force gauge. The force gauge, connected to the pusher, advanced the stent through the porcine urinary tract, allowing measurement of the frictional force required for stent insertion (Fig. 3).

Static friction force *(F*_*s*_*)* was defined as the force required to initiate relative motion between the stent and the opposing contact surfaces (ureteral wall and guidewire). The Arduino was programmed to gradually increase the motor pulling force, and the maximum force recorded by the digital force gauge at the onset of stent movement was recorded as *F*_*s*_. Kinetic friction force (*F*_*k*_) was defined as the resistive force encountered when the stent and the opposing contact surfaces were in relative motion. Following *F*_*s*_ measurement, the Arduino was programmed to maintain a constant pulling velocity. The stable force reading on the digital force gauge as the stent moved continuously was recorded as *F*_*k*_.

To empirically verify constant velocity during *F*_*k*_ measurements, the entire experimental procedure was video recorded. A measuring tape was placed adjacent to the pulling line connected to the digital force gauge and kept within the video frame. Video analysis involved segmenting the footage into equal distance intervals and confirming that the time required to traverse each interval remained consistent throughout the pull.

For each experimental outcome, a linear mixed-effects model was used to evaluate differences among stent types while accounting for repeated measurements within ureter specimens. Stent type and trial were included as fixed effects, and ureter specimen was included as a random intercept. Pairwise comparisons between stent types were adjusted using the Holm method. Statistical significance was defined as *p* < 0.05.

## Results

Linear mixed-effects models demonstrated that stent type was a highly significant predictor of friction after accounting for repeated measurements within ureter specimens. Significant differences among stent types were observed for static friction during the submerged guidewire friction trial (χ²(4) = 274.80, *p* < 0.001), kinetic friction during the submerged guidewire friction trial (χ²(4) = 284.51, *p* < 0.001), static friction during the stent removal trial (χ²(4) = 215.97, *p* < 0.001), kinetic friction during the stent removal trial (χ²(4) = 237.94, *p* < 0.001), static friction during the stent insertion trial (χ²(4) = 292.90, *p* < 0.001), and kinetic friction during the stent insertion trial (χ²(4) = 290.06, *p* < 0.001). Holm-adjusted pairwise comparisons remained statistically significant for all stent comparisons.

Mean *F*_*s*_ values for each stent on a submerged guidewire are summarized in Table 1. Z2 demonstrated the lowest *F*_*s*_ (0.0198 *N* ± 0.003). BS had the highest *F*_*s*_ (7,886.87% greater), while US had the second-lowest *F*_*s*_, measuring 44.44% higher. Mean *F*_*k*_ values on a submerged guidewire are shown in Table 2. Z2 again demonstrated the lowest *F*_*k*_ (0.015 *N* ± 0.002). BS had the highest *F*_*k*_ (10,301.33% greater), while US had the second-lowest *F*_*k*_ (66.67% higher).

**Table 1:** Static friction (*F*_*s*_) measured during the submerged guidewire friction trial. Mean static friction (± SD, 95% confidence intervals) for each stent. Descriptive differences between stents are reported as mean differences (Δ) and percent differences

	BS	Z1	Z2	BB	US
Mean F_s_	1.5812 *N* ± 0.006 (1.5740–1.5884)	0.0566 *N* ± 0.004 (0.0519–0.0613)	0.0198 *N* ± 0.003 (0.0160–0.0236)	0.0360 *N* ± 0.002 (0.0340–0.0380)	0.0286 *N* ± 0.001 (0.0272-0.0300)
BS		Δ = -1.525 -96.42%	Δ = -1.561 -98.75%	Δ = -1.545 -97.72%	Δ = -1.553 -98.19%
Z1	Δ = +1.525 + 2,693.99%		Δ = -0.037 -65.02%	Δ = -0.021 -36.40%	Δ = -0.028 -49.47%
Z2	Δ = +1.561 + 7,886.87%	Δ = +0.037 + 185.86%		Δ = +0.016 + 81.82%	Δ = +0.009 + 44.44%
BB	Δ = +1.545 + 4,292.22%	Δ = +0.021 + 57.22%	Δ = -0.016 -45.00%		Δ = -0.007 -20.56%
US	Δ = +1.553 + 5,428.67%	Δ = +0.028 + 97.90%	Δ = -0.009 -30.77%	Δ = +0.007 + 25.87%	

Fs = static friction; BS = bare silicone stent without coating; Z1 = single outer−coated zwitterionic silicone stent; Z2 = double−coated (inner and outer) zwitterionic silicone stent; BB = Black Silicone Filiform Stent (“Black Beauty”); US = Universa Soft stent; Δ = difference in mean static friction (column mean – row mean), expressed in Newtons (N)

**Table 2:** Kinetic friction (*F*_*k*_) measured during the submerged guidewire friction trial. Mean kinetic friction (± SD, 95% confidence intervals) for each stent. Descriptive differences between stents are reported as mean differences (Δ) and percent differences

	BS	Z1	Z2	BB	US
Mean F_k_	1.5602 *N* ± 0.005 (1.5536–1.5668)	0.0336 *N* ± 0.002 (0.0306–0.0366)	0.0150 *N* ± 0.002 (0.0125–0.0175)	0.0282 *N* ± 0.001 (0.0266–0.0298)	0.0250 *N* ± 0.002 (0.0230–0.0270)
BS		Δ = -1.527 -97.85%	Δ = -1.545 -99.04%	Δ = -1.532 -98.19%	Δ = -1.535 -98.40%
Z1	Δ = +1.527 + 4,543.45%		Δ = -0.019 -55.36%	Δ = -0.005 -16.07%	Δ = -0.009 -25.60%
Z2	Δ = +1.545 + 10,301.33%	Δ = +0.019 + 124.00%		Δ = +0.013 + 88.00%	Δ = +0.010 + 66.67%
BB	Δ = +1.532 + 5,431.91%	Δ = +0.005 + 19.15%	Δ = -0.013 -46.81%		Δ = -0.003 -11.35%
US	Δ = +1.535 + 6,140.80%	Δ = +0.009 + 34.40%	Δ = -0.010 -40.00%	Δ = +0.003 + 12.80%	

Fk = kinetic friction; BS = bare silicone stent without coating; Z1 = single outer−coated zwitterionic silicone stent; Z2 = double−coated (inner and outer) zwitterionic silicone stent; BB = Black Silicone Filiform Stent (“Black Beauty”); US = Universa Soft stent; Δ = difference in mean kinetic friction (column mean – row mean), expressed in Newtons (N)

Mean *F*_*s*_ values during stent removal through the porcine urinary tract are summarized in Table 3. Z2 demonstrated the lowest *F*_*s*_ (0.1616 *N* ± 0.005). BS had the highest *F*_*s*_ (1,002.23% greater), while Z1 had the second-lowest *F*_*s*_ (27.60% higher). Mean *F*_*k*_ values during stent removal are shown in Table 4. Z2 again demonstrated the lowest *F*_*k*_ (0.142 *N* ± 0.005). BS had the highest *F*_*k*_ (1,120.42% greater), while Z1 had the second-lowest *F*_*k*_ (32.96% higher).

**Table 3:** Static friction (*F*_*s*_) measured during the stent removal trial. Mean static friction (± SD, 95% confidence intervals) for each stent. Descriptive differences between stents are reported as mean differences (Δ) and percent differences

	BS	Z1	Z2	BB	US
Mean F_k_	1.7812 *N* ± 0.014 (1.7642–1.7982)	0.2062 *N* ± 0.012 (0.1914–0.2210)	0.1616 *N* ± 0.005 (0.1549–0.1683)	0.3546 *N* ± 0.008 (0.3445–0.3647)	0.3754 *N* ± 0.009 (0.3640–0.3868)
BS		Δ = -1.575 -88.42%	Δ = -1.620 -90.93%	Δ = -1.427 -80.09%	Δ = -1.406 -78.92%
Z1	Δ = +1.575 + 763.82%		Δ = -0.045 -21.63%	Δ = +0.148 + 71.67%	Δ = +0.169 + 82.06%
Z2	Δ = +1.620 + 1,002.23%	Δ = +0.045 + 27.60%		Δ = +0.193 + 119.43%	Δ = +0.214 + 132.30%
BB	Δ = +1.427 + 402.31%	Δ = -0.148 -41.85%	Δ = -0.193 -54.43%		Δ = +0.021 + 5.87%
US	Δ = +1.406 + 374.48%	Δ = -0.169 -45.07%	Δ = -0.214 -56.95%	Δ = -0.021 -5.54%	

Fs = static friction; BS = bare silicone stent without coating; Z1 = single outer−coated zwitterionic silicone stent; Z2 = double−coated (inner and outer) zwitterionic silicone stent; BB = Black Silicone Filiform Stent (“Black Beauty”); US = Universa Soft stent; Δ = difference in mean static friction (column mean – row mean), expressed in Newtons (N)

**Table 4:** Kinetic friction (*F*_*k*_) measured during the stent removal trial. Mean kinetic friction (± SD, 95% confidence intervals) for each stent. Descriptive differences between stents are reported as mean differences (Δ) and percent differences

	BS	Z1	Z2	BB	US
Mean F_k_	1.7330 *N* ± 0.009 (1.7223–1.7437)	0.1888 *N* ± 0.002 (0.1861–0.1915)	0.1420 *N* ± 0.005 (0.1364–0.1456)	0.3418 *N* ± 0.007 (0.3336–0.3509)	0.3554 *N* ± 0.009 (0.3546–0.3762)
BS		Δ = -1.544 -89.11%	Δ = -1.591 -91.81%	Δ = -1.391 -80.28%	Δ = -1.378 -79.49%
Z1	Δ = +1.544 + 817.90%		Δ = -0.047 -24.79%	Δ = +0.153 + 81.04%	Δ = +0.167 + 88.24%
Z2	Δ = +1.591 + 1,120.42%	Δ = +0.047 + 32.96%		Δ = +0.200 + 140.70%	Δ = +0.213 + 150.28%
BB	Δ = +1.391 + 407.02%	Δ = -0.153 -44.76%	Δ =-0.200 -58.46%		Δ = +0.014 + 3.98%
US	Δ = +1.378 + 387.62%	Δ = -0.167 -46.88%	Δ = -0.213 -60.05%	Δ = -0.014 -3.83%	

Fk = kinetic friction; BS = bare silicone stent without coating; Z1 = single outer−coated zwitterionic silicone stent; Z2 = double−coated (inner and outer) zwitterionic silicone stent; BB = Black Silicone Filiform Stent (“Black Beauty”); US = Universa Soft stent; Δ = difference in mean kinetic friction (column mean – row mean), expressed in Newtons (N)

Mean *F*_*s*_ values during stent insertion through the porcine urinary tract are summarized in Table 5. Z2 demonstrated the lowest *F*_*s*_ (0.833 *N* ± 0.001). BS had the highest *F*_*s*_ (252.94% greater), while Z1 had the second-lowest *F*_*s*_ (49.34% higher). Mean *F*_*k*_ values during stent insertion are shown in Table 6. Z2 again demonstrated the lowest *F*_*k*_ (0.8154 *N* ± 0.002). BS had the highest *F*_*k*_ (246.82% greater), while Z1 had the second-lowest *F*_*k*_ (50.90% higher).

**Table 5:** Static friction (*F*_*s*_) measured during the stent insertion trial. Mean static friction (± SD, 95% confidence intervals) for each stent. Descriptive differences between stents are reported as mean differences (Δ) and percent differences

	BS	Z1	Z2	BB	US
Mean F_s_	2.94 *N* ± 0.001 (2.9369–2.9431)	1.244 *N* ± 0.001 (1.2406–1.2474)	0.833 *N* ± 0.001 (0.8310–0.8350)	1.461 *N* ± 0.002 (1.4564–1.4656)	1.4854 *N* ± 0.001 (1.4825–1.4883)
BS		Δ = -1.696 -57.69%	Δ = -2.107 -71.67%	Δ = -1.479 -50.31%	Δ = -1.455 -49.48%
Z1	Δ = +1.696 + 136.33%		Δ = -0.411 -33.04%	Δ = +0.217 + 17.44%	Δ = +0.241 + 19.41%
Z2	Δ = +2.107 + 252.94%	Δ = +0.411 + 49.34%		Δ = +0.628 + 75.39%	Δ = +0.652 + 78.32%
BB	Δ = +1.479 + 101.23%	Δ = -0.217 -14.85%	Δ = -0.628 -42.98%		Δ = +0.024 + 1.67%
US	Δ = +1.455 + 97.92%	Δ = -0.241 -16.25%	Δ = -0.652 -43.92%	Δ = -0.024 -1.64%	

Fs = static friction; BS = bare silicone stent without coating; Z1 = single outer−coated zwitterionic silicone stent; Z2 = double−coated (inner and outer) zwitterionic silicone stent; BB = Black Silicone Filiform Stent (“Black Beauty”); US = Universa Soft stent; Δ = difference in mean static friction (column mean – row mean), expressed in Newtons (N)

**Table 6:** Kinetic friction (*F*_*k*_) measured during the stent insertion trial. Mean kinetic friction (± SD, 95% confidence intervals) for each stent. Descriptive differences between stents are reported as mean differences (Δ) and percent differences

	BS	Z1	Z2	BB	US
Mean F_k_	2.828 *N* ± 0.003 (2.8249–2.8311)	1.2304 *N* ± 0.002 (1.2278–1.2330)	0.8154 *N* ± 0.002 (0.8125–0.8183)	1.438 *N* ± 0.004 (1.4327–1.4433)	1.4598 *N* ± 0.002 (1.4574–1.4622)
BS		Δ = -1.598 -56.49%	Δ = -2.013 -71.17%	Δ = -1.390 -49.15%	Δ = -1.368 -48.38%
Z1	Δ = +1.598 + 129.84%		Δ = -0.415 -33.73%	Δ = +0.208 + 16.87%	Δ = +0.229 + 18.64%
Z2	Δ = +2.013 + 246.82%	Δ = +0.415 + 50.90%		Δ = +0.623 + 76.35%	Δ = +0.644 + 79.03%
BB	Δ = +1.390 + 96.66%	Δ = -0.208 -14.44%	Δ = -0.623 -43.40%		Δ = +0.022 + 1.52%
US	Δ = +1.368 + 93.73%	Δ = -0.229 -15.71%	Δ = -0.644 -44.14%	Δ = -0.022 -1.49%	

Fk = kinetic friction; BS = bare silicone stent without coating; Z1 = single outer−coated zwitterionic silicone stent; Z2 = double−coated (inner and outer) zwitterionic silicone stent; BB = Black Silicone Filiform Stent (“Black Beauty”); US = Universa Soft stent; Δ = difference in mean kinetic friction (column mean – row mean), expressed in Newtons (N)

## Discussion

The reductions in *F*_*s*_ and *F*_*k*_ observed with zwitterionic-coated silicone stents have important implications for stent-tissue interactions. High-friction stent materials are associated with increased insertion difficulty and patient discomfort [4]. Surface-urine interactions following stent placement lead to rapid conditioning-film formation, which facilitates bacterial adhesion, biofilm development, and mineral deposition [4]. In the urinary tract, infectious encrustation is predominantly composed of hydroxyapatite and struvite induced by urease-positive bacteria such as *Proteus mirabilis* [12].

In this benchtop study, the double-coated (inner and outer) zwitterionic silicone stent (Z2) consistently demonstrated the lowest *Fs* and *F*_*k*_ when compared with bare silicone stent without coating (BS), a single outer-coated zwitterionic silicone stent (Z1), Black Silicone Filiform stent (“Black Beauty”) (BB), and a Universa Soft stent (US) across all three experimental configurations: submerged guidewire, porcine ureteral removal, and porcine ureteral insertion. These findings were supported by linear mixed-effects modeling, which demonstrated a significant effect of stent type across all six friction outcomes after accounting for repeated measurements within ureter specimens. The consistent reductions in friction across all three experimental configurations support the potential of zwitterionic surface modification as a friction-lowering strategy across different experimental models and testing geometries.

Differences in frictional performance between Z1 and US stent across experimental models likely reflect differences in the dominant frictional interfaces being tested. In the submerged guidewire model, friction was isolated to the internal stent–guidewire interface. Because the Z1 stent incorporates a zwitterionic coating only on its external surface, its internal lumen remains unmodified, whereas the Z2 stent includes both internal and external zwitterionic coatings. As a result, Z2 demonstrated lower friction than Z1 in this model, while US exhibited lower friction than Z1 despite the absence of a zwitterionic coating. In contrast, in the porcine ureteral insertion and removal models, friction arises from both internal stent–guidewire interactions and external stent–ureteral wall contact. Under these conditions, the external zwitterionic coating of Z1 becomes functionally relevant, resulting in lower friction compared with US.

Zwitterionic polymers are particularly attractive because their balanced positive and negative charges promote formation of a tightly bound hydration layer. In in vitro urinary device studies, this lubricative interface has been shown to reduce bacterial adhesion and crystal encrustation while improving surface lubricity [10]. Hydrophilic coatings similarly prevent protein and bacterial adhesion through water-binding mechanisms that form low-friction, hydrated surfaces [13]. Prior surface-modification strategies for ureteral stents have leveraged hydrophilic surface layers that bind water molecules to form a hydration layer, improving lubricity and reducing bacterial adhesion and mineral-salt deposition [14].

However, many hydrophilic and antifouling coatings require surface activation steps to ensure stable and durable attachment on inert polymeric substrates, highlighting the need for surface chemistries that maintain low fouling and lubricity while remaining robust under clinically relevant mechanical and environmental conditions [8, 15]. Zwitterionic polymers, when stably integrated onto silicone surfaces, may help address this gap by sustaining a strongly bound hydration layer capable of resisting protein adsorption and bacterial attachment. Silicone provides a favorable base material for urinary stents, with advantages including softness and improved patient comfort compared with polyurethane stents [7]. Building on this substrate, zwitterionic coatings have been evaluated across multiple urinary devices. On silicone urinary catheters, sulfobetaine-based zwitterionic coatings significantly reduce biofilm formation and bacterial adherence relative to unmodified silicone [9]. A bilayer polyzwitterionic hydrogel coating has also been demonstrated on polydimethylsiloxane (PDMS) silicone and other biomedical substrates, producing a stable hydrophilic, low-friction, and antifouling surface that resists nonspecific protein adsorption and reduces cell and platelet adhesion [16]. More recently, a zwitterionic multifunctional coating applied to silicone urinary devices has demonstrated suppression of bacterial attachment and crystalline deposition under dynamic urine environments, supporting their potential to mitigate biofilm formation and encrustation during indwelling use [10].

A recent benchtop study evaluated zwitterionic polysulfobetaine–coated silicone stents in a supraphysiologic artificial urine model inoculated with urease-positive *Proteus mirabilis*, demonstrating reduced stent encrustation compared with bare silicone and a commercially available silicone stent [11]. However, this study focused exclusively on mass-based encrustation outcomes using suspended stent segments and did not assess frictional behavior, guidewire-assisted insertion or removal, or interaction with ureteral tissue. To our knowledge, no prior studies have directly quantified static or kinetic friction of zwitterionic-coated silicone ureteral stents under tissue-mimicking conditions. The present study therefore represents the first evaluation of zwitterionic-coated silicone stent friction in models relevant to clinical ureteral stent placement and removal.

Despite these encouraging findings, applying durable zwitterionic coatings to silicone has been technically challenging. Medical-grade silicone is chemically inert and highly hydrophobic, often requiring surface activation strategies to enable stable attachment of hydrophilic or zwitterionic coatings [17]. To overcome this, surface activation strategies such as plasma treatments are commonly used to create reactive adhesion layers for zwitterionic coatings [18–20]. The requirement for an intermediate surface-functionalization step highlights the technical complexity of applying zwitterionic coatings to silicone stent surfaces [18–20].

Recent advances in surface-activation chemistry have improved the stability of hydrophilic and antifouling coatings on silicone substrates. Historically, zwitterionic coatings have been limited by complex, multi-step fabrication processes that hinder scalability. In contrast, the zwitterionic coating evaluated in this study can be applied using a simplified approach that supports reproducible manufacturing and provides clinically relevant reductions in friction during stent insertion and removal.

Cook Black Silicone stents have demonstrated the lowest mineral deposition in lithogenic artificial-urine encrustation models, outperforming other commercially available stent materials in comparative in vitro assays [21]. In this context, our findings suggest that zwitterionic-coated silicone stents may extend the advantages of silicone-based stents by preserving their favorable encrustation profile while markedly reducing both *F*_*s*_ and *F*_*k*_. Notably, friction was substantially lower not only compared with bare silicone but also compared with widely used commercial comparators, including Black Beauty and Universa Soft, suggesting that zwitterionic modification overcomes silicone’s longstanding limitation of high surface friction while retaining its biocompatibility.

In clinical practice, stents with high friction can be more difficult to advance over a guidewire, often requiring greater pushing force and potentially contributing to increased ureteral wall irritation or trauma, especially in tortuous or narrowed ureters. Conversely, smoother, more lubricious stents may reduce insertion forces and procedural difficulty. By combining low friction with an inherently low-encrustation material, zwitterionic-coated silicone stents may improve procedural handling and reduce long-term stent-related complications.

This study has several limitations. As a benchtop investigation using a submerged guidewire model and ex vivo porcine ureters, it cannot fully reproduce in vivo ureteral biomechanics, variable insertion angles, tissue deformation, guidewire variability, operator-dependent movements, or biologic responses. However, the use of a benchtop model allowed us to control all these variables to ensure reproducible comparisons. To facilitate stent placement, both the bladder and kidney were opened. It is possible that opening the bladder and kidney might have a subtle effect on native ureteral biomechanics, tissue tension, and physiologic pressure conditions. Only one stent diameter and length from each material category and only one silicone and one polyurethane stent were evaluated; therefore, it remains unknown whether similar reductions in friction would be observed with alternative stent diameters, lengths, or designs. The BB stent was selected because it is one of the most established commercially available silicone ureteral stents, while the US stent was selected since this is the primary stent utilized in our institution. Additionally, friction was measured over short intervals, and long-term coating durability, biofilm formation, encrustation, and clinical outcomes were not evaluated. Because repeated trials were performed using the same ureteral segments, progressive mechanical alterations to the tissue, including stretching, surface abrasion, or loss of mucosal integrity, may have influenced friction measurements across successive trials. Although the order of stent testing within each renal unit was randomized to minimize systematic order effects, potential carry-over effects from repeated tissue use cannot be excluded.

While the present study demonstrated consistent reductions in friction under controlled ex vivo conditions, these findings will need to be confirmed in clinical series. In addition, whether reduced friction will result in improved patient comfort will also need to be studied clinically. Taken together, these findings suggest that zwitterionic surface modification may address a longstanding trade-off in ureteral stent design by combining the favorable biocompatibility and encrustation resistance of silicone with markedly reduced surface friction.

## Conclusions

The double-coated (inner and outer) zwitterionic silicone stent (Z2) consistently demonstrated the lowest static and kinetic friction forces across all benchtop models. By substantially lowering friction, zwitterionic-coated silicone stents may reduce insertion forces, and their use could therefore improve procedural ease and patient comfort relative to currently available stent designs.

## Data Availability

The datasets generated and analyzed in the study are available from the corresponding author on reasonable request.

## Abbreviations

UTIs: urinary tract infections
BS: bare silicone stent without coating
Z1: single outer-coated zwitterionic silicone stent
Z2: double-coated (inner and outer) zwitterionic silicone stent
BB: Black Silicone Filiform stent (“Black Beauty”)
US: Universa Soft stent
UVJ: ureterovesical junction
UPJ: ureteropelvic junction
F_s_: static friction
F_k_: kinetic friction
